# Development of selective blockers for Ca^2+^-activated Cl^- ^channel using *Xenopus laevis *oocytes with an improved drug screening strategy

**DOI:** 10.1186/1756-6606-1-14

**Published:** 2008-10-29

**Authors:** Soo-Jin Oh, Jung Hwan Park, Sungyu Han, Jae Kyun Lee, Eun Joo Roh, C Justin Lee

**Affiliations:** 1 Center for Neural Science, Future Fusion Technology Laboratory, Korea Institute of Science and Technology (KIST), Seoul 136-791, Republic of Korea; 2 Center for Chemoinformatics Research, Life Sciences Division, Korea Institute of Science and Technology (KIST), Seoul 136-791, Republic of Korea; 3 Department of Cell and Developmental Biology, Dental Research Institute, School of Dentistry, Seoul National University, Seoul 110-740, Republic of Korea

## Abstract

**Background:**

Ca^2+^-activated Cl^- ^channels (CaCCs) participate in many important physiological processes. However, the lack of effective and selective blockers has hindered the study of these channels, mostly due to the lack of good assay system. Here, we have developed a reliable drug screening method for better blockers of CaCCs, using the endogeneous CaCCs in *Xenopus laevis *oocytes and two-electrode voltage-clamp (TEVC) technique.

**Results:**

Oocytes were prepared with a treatment of Ca^2+ ^ionophore, which was followed by a treatment of thapsigargin which depletes Ca^2+ ^stores to eliminate any contribution of Ca^2+ ^release. TEVC was performed with micropipette containing chelerythrine to prevent PKC dependent run-up or run-down. Under these conditions, Ca^2+^-activated Cl^- ^currents induced by bath application of Ca^2+ ^to oocytes showed stable peak amplitude when repetitively activated, allowing us to test several concentrations of a test compound from one oocyte. Inhibitory activities of commercially available blockers and synthesized anthranilic acid derivatives were tested using this method. As a result, newly synthesized *N*-(4-trifluoromethylphenyl)anthranilic acid with trifluoromethyl group (-CF_3_) at *para *position on the benzene ring showed the lowest IC_50_.

**Conclusion:**

Our results provide an optimal drug screening strategy suitable for high throughput screening, and propose *N*-(4-trifluoromethylphenyl)anthranilic acid as an improved CaCC blocker.

## Background

Ca^2+^-activated Cl^- ^channels (CaCCs) are anion-selective channels that can be activated by an increase in cytosolic Ca^2+^. CaCCs serve a number of important physiological roles in a variety of cell types. These functions include vascular tone regulation, cardiac excitability, smooth muscle contraction, fast block of polyspermy in certain eggs [1]. CaCCs are also known to regulate epithelial secretion of electrolytes and water in kidneys, airways, intestines, pancreas and salivary glands [1]. In addition, CaCCs appear to participate in signal processing of olfactory transduction, photo receptor light response, gustation and somaesthetic sensation by regulating neuronal cell excitability. CaCC currents in non-sensory neurons of the spinal cord and the autonomic nervous system were also reported, and further investigation may prove an even more extensive expression in the nervous system [2]. Despite this physiological importance of CaCC, the channel remains poorly understood at the molecular, biophysical and pharmacological level, owing to the lack of specific pharmacologic tools with high potent and selectivity. Currently available blockers require high concentrations to completely block CaCCs and are known to cause undesirable side effects and block other channels. For example, niflumic acid and 4,4'-diisothiocyanatostilbene-2,2'-disulphonic acid (DIDS) which are widely used to block CaCC also block volume-regulated anion channel (VRAC) in some cell types [3,4]. Niflumic acid, flufenamic acid and 5-nitro-2-(3-phenylpropylamino)-benzoic acid (NPPB) are shown to have a blocking effect on K^+ ^channel current [5,6]. Niflumic acid, flufenamic acid and NPPB also cause an increase in intracellular Ca^2+ ^concentration ([Ca^2+^]_i_) in several cell types, which could elicit other cellular responses [7-11]. Therefore, due to these problems with low potency and selectivity, there is an eminent need for development of better blockers for CaCCs.

*Xenopus laevis *oocytes have been used widely in the field of electrophysiology to study the structure and the function of numerous ion channels and to screen for selective blockers. These oocytes express several native ion channels including CaCC in the plasma membrane [12], which are normally avoided when studying other ion channels by substituting Ca^2+ ^with Ba^2+ ^in the extracellular solution in attempt to prevent activation of endogenous CaCCs. These CaCCs have similar properties in many ways to those in cardiac muscle, smooth muscle, secretory epithelial cells and neurons [13]. There have been a numerous attempts to discover chemical compounds to block the endogenous CaCCs in *Xenopus laevis *oocytes using TEVC (two-electrode voltage clamp) technique. These studies reported half maximal inhibition concentration (IC_50_) of various compounds, including niflumic acid (17 μM of, IC_50_) [14], flufenamic acid (28 μM of IC_50_) [14], DIDS (48 μM of IC_50_) [13], diphenylamine-2-carboxylate (DPC, 111 μM of IC_50_) [13], 9-anthracene carboxylic acid (9-AC, 10.3 μM of IC_50_) [13] and NPPB (22–68 μM of IC_50_) [15]. But the potency of these blockers is relatively low. In other studies, CaCC current was evoked by direct intracellular injection of Ca^2+ ^into an oocyte [12,16] or by depolarizing the membrane which allows Ca^2+ ^entry through voltage gated Ca^2+ ^channels [17-19]. However, these approaches can introduce complications of unstable baseline current due to irregular amplification of Ca^2+ ^concentration upon Ca^2+ ^entry, unpredictable contribution of Ca^2+ ^release from intracellular stores and time dependent inactivation of CaCCs, making it difficult to perform large scale drug screening. Based on the observation that no reliable method of drug screening and no ideal blocker of CaCC in *Xenopus laevis *oocytes have yet been described, we sought to design an optimized experimental protocol ideal for large scale drug screening to find better blockers for CaCCs.

## Results

### Cl^- ^current elicited by Ca^2+ ^influx in oocytes permeabilized with ionomycin

**Figure 1:**
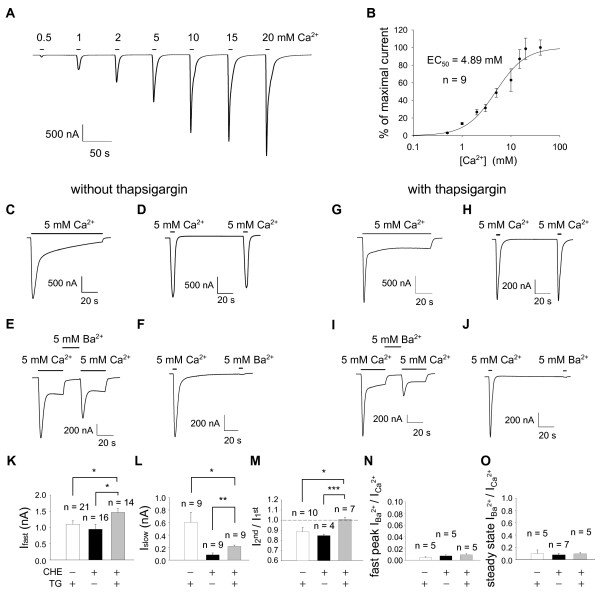
**Endogeneous Ca^2+^ activated Cl- channels in *Xenopus laevis* oocyte.** (A) Currents induced by extracellular Ca^2+^ in a dose dependent manner on ionomycin treated oocyte. (B) Dose response and EC_50_ of Ca^2+^ obtained from (A). (C~F) Currents recorded after treatment of ionomycin without thapsigargin treatment. (C, G) Fast peak and slow component during Ca^2+^ applications. (G~J) Currents recorded after treatment of ionomycin followed by thapsigargin. (D, H) Second application of Ca^2+^ induces slightly reduced fast peak amplitude compared to the first peak. (E, I) Ba^2+^ does not induce the slow component. (F, J) Ba^2+^ does not induce the fast peak. (K~O) Comparison of currents under each condition. CHE+ means that current was measured with chelerythrine added intracellular solution. TG+ indicates that thapsigargin was treated on ionomycin pretreated oocytes. (K) Fast peak amplitude. (L) Slow component amplitude. (M) Summary of the experiments shown in (D) and (H); Ratio of amplitude induced by the first and the second Ca^2+^. (N) Summary of the experiments shown in (E) and (I). (O) Summary of the experiments shown in (F) and (J). n indicates number of oocytes. Error bars indicate SEMs. * indicates statistically significant difference by two-tailed t-test. *, *p* < 0.05; **, *p* < 0.01; ***, *p* < 0.001

To find optimal conditions for drug screening using CaCCs in *Xenopus *oocytes, we first characterized the CaCC currents in *Xenopus *oocytes and compared various treatment conditions. To permeablize the oocyte membrane to allow Ca^2+ ^influx, oocytes were treated with 10 μM ionomycin for 30 min in Ca^2+ ^free solution. In oocytes, Ca^2+ ^entry and activation of an inward current were achieved by switching from Ca^2+ ^free external solution to Ca^2+ ^containing solution [20]. After membrane permeabilization, application of external Ca^2+ ^evoked Cl^- ^currents consisting of fast peak (I_fast_) and slow steady state component (I_slow_, Figure 1C) [20]. The fast component was elicited by 5 s exposure to extracellular Ca^2+ ^([Ca^2+^]_o_) in a dose dependant manner (Figure 1A) and calculated EC_50 _for [Ca^2+^]_o _was 4.89 mM (n = 9) (Figure 1B). Therefore, 5 mM [Ca^2+^]_o _was used to induce Cl^- ^current in all subsequent experiments. I_fast _and I_slow _were not induced by substituting Ca^2+ ^with Ba^2+ ^in each condition (Figure 1E, F, I, J, N, O), indicating that both of these currents were activated by Ca^2+ ^entry.

In response to repetitive 5 s applications of the same dose of [Ca^2+^]_o_, the amplitude of 2^nd ^response was smaller compared to the initial response (I_fast_^2nd^/I_fast_^1st ^= 0.89 ± 0.04, Figure 1M, white bar), most likely due to an activation of a Ca^2+^-dependent protein kinase C (PKC) [21]. To exclude the effect of PKC in CaCC current, PKC inhibitor chelerythrine was added to the intracellular solution. Inclusion of chelerythrine decreased the variability represented by the standard error of mean value (I_fast_^2nd^/I_fast_^1st ^= 0.85 ± 0.013, Figure 1M, black bar), but still the 2^nd ^response remained smaller than the 1^st ^response (Figure 1D). Additional variability in peak amplitude can come from the Ca^2+ ^induced Ca^2+ ^release from intracellular stores. To eliminate the contribution of Ca^2+ ^release from intracellular stores, Ca^2+ ^ATPase inhibitor, thapsigargin was treated on ionomycin pretreated oocytes. Under the condition of ionomycin treatment followed by thapsigargin treatment and recording with chelerythrine added intracellular solution, the peak amplitudes of two consecutive responses to 5 mM external Ca^2+ ^were almost the same with relatively low standard error of mean value (Figure 1H, I_fast_^2nd^/I_fast_^1st ^= 1.01 ± 0.02 (n = 7), Figure 1M, gray bar). Therefore, a reliable protocol for drug screening of CaCCs in *Xenopus *oocytes was established with treatment of 1 μM thapsigargin for 90 min to ionomycin treated oocytes followed by recording with microelectrodes filled with intracellular solution containing 1 μM chelerythrine.

### Effect of known blockers and commercially available chemical compounds on CaCC in Xenopus oocytes

**Figure 2:**
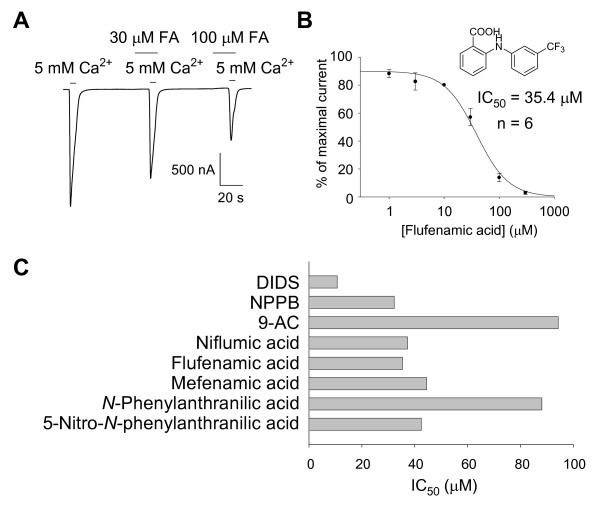
**Effect of known blockers on Ca^2+ ^activated Cl^- ^channel**. (A) Trace of Ca^2+ ^activated Cl^- ^channel current before and during application of flufenamic acid (FA). (B) Dose response relation of flufenamic acid block of Ca^2+ ^activated Cl^- ^current. (C) Summary of IC_50_s of commercially available blockers for Ca^2+^-activated Cl^- ^channel. n indicates number of oocytes. Error bars indicate SEMs.

**Figure 3:**
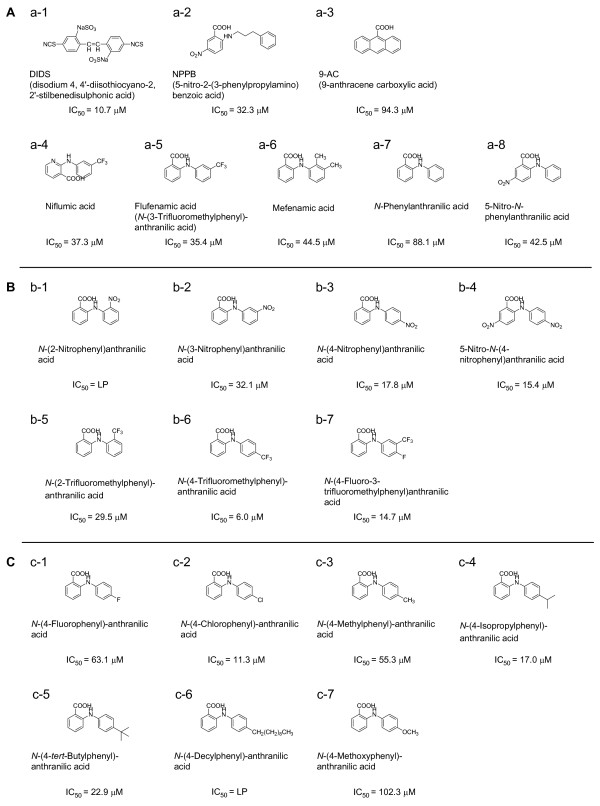
**Chemical structures and IC_50_s of known blockers and anthranilic acid derivatives**. (A) Known blockers. (B) Anthranilic acid derivatives; positional compounds. (C) Anthranilic acid derivatives that have variable substituent group on *para *position of benzene ring. LP: Low Potency. IC_50 _> 200 μM. n indicates number of oocytes.

Using the optimized drug screening protocol, the effect of known blockers of CaCC was tested. Over the concentration range tested (1 μM – 300 μM), each of the typical CaCC blockers caused a concentration dependent block of CaCC currents (Figure 2A) and IC_50_s were obtained from the dose-response curves (Figure 2B). The name and structure for each chemical compound are listed in Figure 3, and each chemical compound was numbered as shown on top of each chemical structure. From the recordings, IC_50_s were found to be 10.7 μM for DIDS, 32.3 μM for NPPB, 94.3 μM for 9-AC, 37.3 μM for niflumic acid, and 35.4 μM for flufenamic acid (Figure 2C, Figure 3A, Table 1). Other blockers generally known for other Cl^- ^channels were also tested under the same condition. IC_50_s were found to be 44.5 μM for mefenamic acid and 88.1 μM for *N*-Phenylanthranilic acid. Except for DIDS, most of known blockers displayed higher IC_50 _values compared to the previously reported values (Table 1).

**Table 1:** IC_50_s of known blockers and anthranilic acid derivatives.

Compound number	Chemical compound	IC_50 _*	IC_50_	n
a-1	DIDS	48	10.7	6
a-2	NPPB (5-nitro-2-(3-phenylpropylamino)benzoic acid)	22–68	32.3	6
a-3	9-AC (9-anthracene carboxylic acid)	10.3	94.3	5
a-4	Niflumic acid	17	37.3	7
a-5	Flufenamic acid (*N*-(3-Trifluoromethylphenyl)anthranilic acid)	28	35.4	6
a-6	Mefenamic acid		44.5	6
a-7	*N*-Phenylanthranilic acid		88.1	6
a-8	5-Nitro-*N*-phenylanthranilic acid		42.5	8
b-1	*N*-(2-Nitrophenyl)anthranilic acid		LP	7
b-2	*N*-(3-Nitrophenyl)anthranilic acid		32.1	7
b-3	*N*-(4-Nitrophenyl)anthranilic acid		17.8	6
b-4	5-Nitro-*N*-(4-nitrophenyl)anthranilic acid		15.4	5
b-5	*N*-(2-Trifluoromethylphenyl)anthranilic acid		29.5	6
b-6	*N*-(4-Trifluoromethylphenyl)anthranilic acid		6.0	6
b-7	*N*-(4-Fluoro-3-trifluoromethylphenyl)anthranilic acid		14.7	6
c-1	*N*-(4-Fluorophenyl)anthranilic acid		63.1	6
c-2	*N*-(4-Chlorophenyl)anthranilic acid		11.3	6
c-3	*N*-(4-Methylphenyl)anthranilic acid		55.3	7
c-4	*N*-(4-Isopropylphenyl)anthranilic acid		17.0	6
c-5	*N*-(4-*tert*-Butylphenyl)anthranilic acid		22.9	7
c-6	*N*-(4-Decylphenyl)anthranilic acid		LP	6
c-7	*N*-(4-Methoxyphenyl)anthranilic acid		102.3	5

IC_50 _* means IC_50 _previously studied. LP: Low Potency. IC_50 _> 200 μM n indicates number of oocytes.

We searched for other commercially available chemical compounds that have similar structure to known blockers. Since flufenamic acid, mefenamic acid and *N*-Phenylanthranilic acid commonly have anthranilic acid backbone, which is composed of two benzene rings, anthranilic acids containing a nitro group (-NO_2_) such as 5-Nitro-*N*-phenylanthranilic acid (a-8), *N*-(2-Nitrophenyl)anthranilic acid (b-1) and *N*-(3-Nitrophenyl)anthranilic acid (b-2) were tested (Figure 3A, B). IC_50_s were 42.5 μM for a-8 and 32.1 μM for b-2. b-1 showed low potency for CaCC block (Figure 3A, B, Table 1). Interestingly, even though a-8, b-1 and b-2 have a similar chemical composition, their blocking effect on CaCC was quite different. The only difference between three chemical compounds is the position of -NO_2_. Therefore, the position of substituent group on benzene ring of the anthranilic acid backbone apparently affected the blocking effect on CaCC.

### Positional effect of substituent group of benzene ring on block of CaCC current

**Figure 4:**
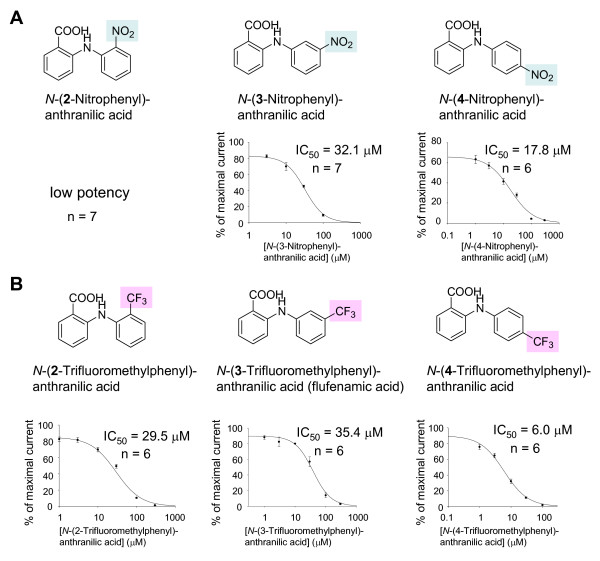
**Positional effect of substituent group on the phenyl ring of blocker that affects block of Ca^2+^-activated Cl^- ^current**. (A) Comparison of chemical structure, IC_50 _and dose response between *N*-(2-nitrophenyl)anthranilic acid, *N*-(3-nitrophenyl)anthranilic acid and *N*-(4-nitrophenyl)anthranilic acid in which the nitro (-NO_2_) group on the benzene ring is positioned at *ortho*, *meta *and *para *position. (B) Comparison of chemical structure, IC_50 _and dose response between flufenamic acid and derivatives *N*-(2-trifluoromethylphenyl)anthranilic acid and *N*-(4-trifluoromethylphenyl)anthranilic acid in which the trifluoromethyl (-CF_3_) group on the benzene ring is positioned at *ortho*, *meta *and *para *position. Shaded boxes indicate the substituent groups tested. n indicates number of oocytes. Error bars indicate SEMs.

To examine the positional effect of -NO_2 _of benzene ring on block of CaCC current in more detail, we synthesized compounds with substitutions on the benzene ring (see additional file 1). We synthesized *N*-(4-nitrophenyl)anthranilic acid (b-3) that has -NO_2 _on its *para *position and tested for the block of CaCC current. This compound showed a significantly improved IC_50 _of 17.8 μM, compared to b-1 that has -NO_2 _on its *ortho *position (IC_50 _> 200 μM) or b-2 that has – NO_2 _on its *meta *position (IC_50_= 32.1 μM Figure 4A). Based on the fact that blocking effects of -NO_2 _on the benzene ring of either side of anthranilic acid backbone (a-8, b-3) showed improved IC_50 _compared to the *N*-Phenylanthranilic acid itself (a-7), we synthesized 5-Nitro-*N*-(4-nitrophenyl)anthranilic acid (b-4) that has -NO_2 _on its *para *position on both benzene rings. Interestingly, IC_50 _of b-4 was further enhanced (IC_50 _= 15.4 μM) than a-8 and b-3, indicating that substituent group on *para *position should enhance the blocking effect.

Since flufenamic acid (*N*-(3-trifluoromethylphenyl)anthranilic acid, a-5) has trifluoromethyl group (-CF_3_) in *meta *position, we hypothesized that shifting -CF_3 _from *meta *position to *ortho *or *para *position would result in changes in IC_50_. We synthesized *N*-(2-trifluoromethylphenyl)anthranilic acid (b-5) that has -CF_3 _on its *ortho *position and *N*-(4-trifluoromethylphenyl)anthranilic acid (b-6) that has -CF_3 _on its *para *position. b-5 showed enhanced IC_50 _(IC_50 _= 29.5 μM) than a-5 (Figure 3B, 4B). In particular, b-6 dramatically enhanced the IC_50 _compared to any other compounds (IC_50_= 6.0 μM, Figure 3B, 4B). These data indicate that changing the position of substituent group on the benzene ring of anthranilic acid backbone to *para *position leads to an increase in blocking activity.

### Comparison of blocking effect about anthranilic acid derivatives on CaCC

Since *para *positioned anthranilic acid derivatives showed higher potency of block, we synthesized more anthranilic acid derivatives that have various substituents on *para *position of the benzene ring and tested on CaCC currents. However, blocking activity of these derivatives was not better than b-5 with -CF_3 _on *para *position as indicated by their IC_50_s: c-2 (chloryl group, -Cl, IC_50 _= 11.3 μM), c-4 (isopropyl group, -C_3_H_7_, IC_50 _= 17.0 μM) and c-5 (butyl group, – C_4_H_9_, IC_50 _= 22.9 μM (Figure 3C, Table 1). In addition, *N*-(4-Fluorophenyl)-anthranilic acid (c-1, IC_50 _= 63.1 μM), *N*-(4-Methylphenyl)-anthranilic acid (c-3, IC_50_= 55.3 μM), *N*-(4-Decylphenyl)-anthranilic acid (c-6, IC_50_> 200 μM) and *N*-(4-Methoxyphenyl)-anthranilic acid (c-7, IC_50 _= 102.3 μM) which have fluoro group (-F), methyl group (-CH_3_), decyl group (-CH_2_(CH_2_)_8_CH_3_) and methoxy group (-OCH_3_) showed relatively low potency for CaCC block, respectively (Figure 3C, Table 1). Therefore, the most potent derivative among the compounds tested was the *N*-(4-trifluoromethylphenyl)anthranilic acid that has -CF_3 _on its *para *position with IC_50 _of 6.0 μM.

## Discussion

Since the lack of useful tools for drug screening has impeded the development of better blocker for CaCCs, we designed an improved drug screening protocol utilizing endogeneous CaCCs in *Xenopus *oocytes. Our newly designed protocol consists of *Xenopus Leavis *oocytes treated with ionomycin and thapsigargin followed by recording with intracellular solution containing chelerythrine. These manipulations provide a consistent induction of CaCC mediated currents with similar amplitude upon repeated application of 5 mM external Ca^2+^. Treatment of ionomycin allows constant entry of Ca^2+ ^from extracellular solution, whereas thapsigargin prevents irregular concentration fluctuations caused by Ca^2+ ^release amplification from internal stores. Elevation of intracellular Ca^2+ ^due to Ca^2+ ^influx through Ca^2+ ^channels formed by ionomycin should cause inactivation of the CaCC conductance via activation of PKC [21]. Thus, inhibition of PKC should decrease the channel modulation by PKC phosphorylation, which was shown to modulate the CaCC current [22]. Thus, intracellular solution containing PKC inhibitor chelerythrine was used for recording of CaCC current to exclude the effect of PKC. Under these conditions, the intracellular Ca^2+ ^concentration was kept constant during induction of CaCC current, and current rundown due to PKC inactivation was kept at minimal level. These manipulations made it possible to measure blocking effect of various compounds on CaCC-mediated current in *Xenopus leavis *oocytes more accurately.

In addition, some blockers such as niflumic acid change the intracellular Ca^2+ ^buffering which causes an increase in intracellular Ca^2+ ^concentration [7-11]. Since Cl^- ^current is induced exclusively by external Ca^2+ ^in our protocol, we were able to isolate the blocking effect of various compounds on channel activities from the effect of compounds on Ca^2+ ^buffering capacity.

Utilizing this optimized protocol, we first compared the effect of known blockers of CaCC. We found that IC_50_s of DIDS, NPPB, 9-AC, niflumic acid and flufenamic acid on CaCC current in *Xenopus *oocytes were 10.7 μM (DIDS), 32.3 μM (NPPB), 94.3 μM (9-AC), 37.3 μM (niflumic acid) and 35.4 μM (flufenamic acid), whereas previously reported values are 48 μM, 28–68 μM, 10.3 μM, 17 μM and 28 μM, respectively (see Table 1). Blocking effect of each known blocker differed from previous reports. It is likely due to differences in our protocol. We suggest that our modified screening protocol provides more exact profile of CaCC blocker on CaCC in *Xenopus Leavis *oocytes due to the reliability and repeatability of our assay protocol. In addition, some blockers that were generally used for other Cl^- ^channels such as mefenamic acid and *N*-Phenylanthranilic acid showed comparable blocking effect on CaCC in *Xenopus Leavis *oocytes with IC_50_s of 44.5 μM and 88.1 μM.

Based on the fact that several known blockers of CaCCs have structural similarity as anthranilic acid which is composed of two benzene rings, we searched for CaCC blocker candidates with the structural similarity among commercially available chemical compounds. Even though, the number of compounds were not enough to explain exact SAR (structure-activity relationship), we could figure out the correlation between biological activity and the kind and position of substituents. We found that 5-Nitro-*N*-phenylanthranilic acid and *N*-(3-Nitrophenyl)anthranilic acid both showed similar level of blocking effect as niflumic acid or flufenamic acid. Interestingly, blocking potency was quite different between *N*-(3-Nitrophenyl)anthranilic and *N*-(2-Nitrophenyl)anthranilic acid even though their chemical composition is identical, except for the relative position of -NO_2 _in the benzene ring. This reflected that the position of -NO_2 _affected the blocking activity to CaCC. Synthesized *N*-(4-Nitrophenyl)anthranilic acid that has -NO_2 _on its *para *position showed improved blocking ability compare to *N*-(3-Nitrophenyl)anthranilic and *N*-(2-Nitrophenyl)anthranilic acid with -NO_2 _on *meta *and *ortho *position, respectively. Likewise, synthesized *N*-(4-trifluoromethylphenyl)anthranilic acid that has -CF_3 _on its *para *position blocked CaCC better than flufenamic acid (*N*-(3-trifluoromethylphenyl)anthranilic acid) and *N*-(2-trifluoromethylphenyl)anthranilic acid with -CF_3 _on *meta *and *ortho *position, respectively. These results suggested that the positioning of substituent group on *para *site contributes to a higher affinity of these compounds to CaCC. Therefore, we concluded that antrhranilic acid derivatives containing *para *positioned substituent group have high potency of CaCC block.

Additionally synthesized chemical compounds are anthranilic acid derivatives that have various substituent groups such as various hydrocarbons introduced into the benzene ring at *para *position. IC_50_s of *N*-(4-Chlorophenyl)-anthranilic acid, *N*-(4-Isopropylphenyl)-anthranilic acid and *N*-(4-*tert*-Butylphenyl)-anthranilic acid were similar in IC_50 _of *N*-(4-Nitrophenyl)anthranilic acid. However, low blocking effect was shown in *N*-(4-Fluorophenyl)-anthranilic acid, *N*-(4-Methylphenyl)-anthranilic acid and *N*-(4-Decylphenyl)-anthranilic acid. These results suggested that there should be no strong correlation between blocking effect and size of substituent group in CaCC blockers. It has been reported that blocker size affects voltage dependence rather than potency of CaCC block because large blockers lodge at sites less deep in the channel [1]. Taken together, *N*-(4-trifluoromethylphenyl)anthranilic acid that has -CF_3 _on its *para *position in one benzene ring is most potent CaCC blocker candidate so far.

Ion channels endogeneously expressed in *Xenopus Leavis *oocytes have extensively used in biological and pharmacological research. CaCCs in *Xenopus Leavis *oocytes have similar properties in many ways to those in other tissue. It has been revealed that several human diseases are involved in CaCCs. However, its molecular identity is not clear yet. Recently, CLCA [23,24], bestrophin [25], tweety [26] and TMEM16A [27] have been proposed as the molecular candidates. It is important to note that blocker candidates confirmed in *Xenopus Leavis *oocytes should be also examined in these candidate molecules and CaCCs in other various tissues. Nevertheless, several common blockers for CaCCs have undesirable side effects: they can affect the cellular Ca^2+ ^level [7-11], and block VRACs [3] and K^+ ^channels [5,6]. Recent study also identified that niflumic acid, flufenamic acid and Indomethacin are non-steroidal anti-inflammatory drug (NSAID) that have inhibitor potencies against both cyclo-oxygenase (COX) 1 and 2 [28]. Considering the structural similarity, other candidates of CaCC blocker may have the similar side effects. Therefore, future experiments should be followed to test whether blocker candidates show decreased side effects.

## Conclusion

This study has shown the development of reliable screening method for CaCC blocker using endogeneous CaCCs in *Xenopus laevis *oocytes. We found that anthranilic acid derivatives containing *para *positioned substituent group have high potency of CaCC block and *N*-(4-trifluoromethylphenyl)anthranilic acid is most effective CaCC blocker among the synthesized chemical compounds.

## Methods

### Preparation of oocytes

Matured stage V–VI oocytes [29] harvested from adult *Xenopus laevis *females (Xenopus I, Michigan, USA) which were maintained in an automated maintenance system, Xenopus System (Aquatic Habitats, Florida, USA). The animals were anaesthetized by cooling with ice [30]. Ovarian follicles were surgically removed, treated with 2 mg/ml collagenase type IA at room temperature for 90 min in Ca^2+ ^free Barth's solution containing 89 mM NaCl, 1.0 mM KCl, 2.4 mM NaHCO_3_, 0.82 mM MgSO_4 _and 10 mM HEPES (pH 7.4). The oocytes were extensively rinsed with normal Barth's solution containing 88 mM NaCl, 1.0 mM KCl, 2.4 mM NaHCO_3_, 0.82 mM MgSO_4_, 0.33 mM Ca(NO_3_)_2_, 1.41 mM CaCl_2 _and 5 mM HEPES (pH 7.4), placed in culture Barth's solution containing 88 mM NaCl, 1.0 mM KCl, 2.4 mM NaHCO_3_, 0.82 mM MgSO_4_, 0.33 mM Ca(NO_3_)_2_, 0.91 mM CaCl_2_, 10 mM HEPES, 10 μg/ml streptomycin and 10 μg/ml penicillin (pH 7.4), and maintained at 18°C. Oocytes were used 1–4 days after isolation.

### Synthesis

All commercially available chemicals were reagent grade and used as purchased unless stated otherwise. All reactions were performed under an inert atmosphere of dry argon or nitrogen using distilled dry solvents. Reactions were monitored by TLC analysis using Merck silica gel 60 F-254 thin layer plates. Flash column chromatography was carried out on Merck silica gel 60 (230–400 mesh) by preparative LC system. ^1^H and ^13^C NMR spectra were recorded either on a spectrometer operating at Bruker 400 and 100 MHz, respectively. Preparations of chemicals are described in detail in Additional file 1.

### Electrophysiology

To permeabilize the membrane to Ca^2+^, oocytes were incubated in the oocyte recording solution containing 96 mM NaCl, 2 mM KCl, 2 mM MgCl_2_, 0.5 mM EGTA and 10 mM HEPES (pH 7.4), added with 10 μM ionomycin for 30 min. The ionomycin was then removed from the external solution by washing with oocyte recording solution. In case of thapsigargin treatment, ionomycin treated oocytes were subsequently incubated in the oocyte recording solution containing 1 μM thapsigargin for 90 min. Then thapsigargin was also washed with the oocyte recording solution. Two electrode voltage-clamp recordings were made using Warner model OC725B two-electrode voltage clamp amplifier (Warner Instruments, Inc., Hamden, CT) with 1 M KCl-filled microelectrodes (WPI; Sarasota, FL; 1B150F-4) pulled with a P-97 programmable pipette puller (Sutter Instruments Co.; Novato, CA). The pipettes had resistances of 1–3 MΩ. During the recording oocytes were continuously perfused with oocyte recording solution. All recordings were from a holding potential of -60 mV. Drugs were prepared in separate bottles and applied by gravity. Flow of solutions was approximately 1 ml/min.

### Chemicals

Chemical compounds including the following reagents were purchased from Sigma-Aldrich Co. (St. Louis, MO, USA); Collagenase type 1A, ionomycin-Ca^2+ ^salt, thapsigargin, chelerythrine. HEPES (N-[2-hydroxyethyl]piperazine-N'-[ethanesulfonic acid]) was obtained from J.T Baker (Mallinckrodt Baker, Inc. Phillipsburg, NJ, USA).

### Analysis of data

Currents were digitally recorded with AxoScope software (Axon Instruments, Burlingame, CA, USA) and data analysis was done with SigmaPlot 10.0 (Systat Software, Inc., CA, USA). All the current responses during a blocker were normalized to the average of a Ca^2+ ^induced Cl^- ^current applied before blocker application. Normalized and average data were fitted to the SigmaPlot's Logistic, 3 Parameter curve to determine dose-response relationship and IC_50_. All data are expressed as mean ± standard error of mean and statistical analysis was performed using a 2-tailed t-test.

## Competing interests

The authors declare that they have no competing interests.

## Authors' contributions

SJO carried out electrophysiological recordings, data analysis and manuscript preparation. JHP, JKL and EJR designed and synthesized chemical compounds. SKH participated in electrophysiological recordings and synthesis of chemical compounds. CJL conceived the idea, coordinated the study, carried our data interpretation and drafted the manuscript. All authors have read and approved the manuscript.

## Supplementary Material

Additional file 1 **Methods for synthesis of anthranilic acid derivatives.** Click here for file
