# Supplementary material for: Development of selective blockers for Ca2+-activated Cl- channel using Xenopus laevis oocytes with an improved drug screening strategy
Source: Mol Brain. 2008 Oct 29;1:14. doi: 10.1186/1756-6606-1-14 (PMC2585076; doi:10.1186/1756-6606-1-14)
Supplement: Additional file 1 — Methods for synthesis of anthranilic acid derivatives. [file 1756-6606-1-14-S1.doc]

**Methods for synthesis of anthranilic acid derivatives**

**Scheme 1 Preparation of 2-bromobenzoic acid methyl ester**

Chlorotrimethylsilane (22.2 ml, 0.174 mol) was added dropwise to a solution of 2-bromobenzoic acid (17.5 g, 0.087 mol) in anhydrous methyl alcohol (150 ml) at 0 ℃. After the addition, the mixture was stirred at reflux for 8 hrs. Then, it was quenched by addition of water (50 ml) and excess methyl alcohol was removed by rota-evaporator. The residue was extracted with ethyl acetate and washed with brine. The combined organic layer was dried over anhydrous MgSO_4_, filtered and the solvent was evaporated to give product (17.8 g, 95 %) as a pale yellow liquid which was used to next step without any further purification.

R*_f_* = 0.72 (EA / n-Hex = 1 : 2)

^1^H NMR (400 MHz, CDCl_3_) δ = 3.93 (s, 3H), 7.29-7.37 (m, 2H), 7.65 (dd, *J* = 1.58 Hz and 1.54 Hz, 1H), 7.78 (dd, *J* = 2.04 Hz and 2.08 Hz, 1H); ^13^C NMR (100 MHz, CDCl_3_) δ = 52.36, 121.53, 127.05, 131.19, 132.01, 132.46, 134.22, 166.50

**Scheme 2 General procedure of *N*-aryl anthranilic acid methyl ester**

Tris(dibenzylideneacetone)dipalladium(0)-chloroform adduct (0.030 mmol) and 1,1’-bis(diphenylphosphino)ferrocene (0.045 mmol) in anhydrous toluene (40 ml) was stirred at room temperature for 30 min. Then, 2-bromobenzoic acid methyl ester (0.01 mol), Cs_2_CO_3_ (0.014 mol) and the aniline derivative (0.012 mol) were added and stirred at reflux for 36 hrs. The reaction mixture was filtered thru celite and concentrated in vacuo. The residue was purified by column chromatography (SiO_2_, ethyl acetate/hexane = 1:6) to afford the product.

**Scheme 3 General procedure of *N*-aryl anthranilic acid**

Lithium hydroxide (0.02 mol) was added to a solution of *N*-aryl anthranilic acid methyl ester (0.01 mol) in THF/MeOH/H_2_O (3:1:1, 40 ml) and stirred at room temperature for 12 hrs. Then, the reaction mixture was acidified by addition of 1N-HCl (aq) until pH = 3. The mixture was extracted with ethyl acetate and washed with brine. The combined organic layer was dried over anhydrous MgSO_4_, filtered and concentrated in vacuo. The solid was washed with hexane and dried to yield the corresponding acid.

***N*-Phenylanthranilic acid**

Yield = 82 %

White solid

^1^H NMR (400 MHz, CDCl_3_) δ = 6.75 (dd, *J* = 7.11 Hz and 7.09 Hz, 1H), 7.13 (quintet, 1H), 7.21-7.39 (m, 6H), 8.05 (d, *J* = 8.04 Hz, 1H), 9.31 (bs, 1H); ^13^C NMR (100 MHz, CDCl_3_) δ = 110.37, 114.02, 117.17, 123.14, 124.10, 129.43, 132.61, 135.23, 140.30, 148.92, 173.84

***N*-(4-Fluorophenyl)anthranilic acid**

Yield = 67 %

White powder

^1^H NMR (400 MHz, CDCl_3_) δ = 6.75 (quintet, 1H), 7.01-7.10 (m, 3H), 7.21-7.39 (m, 6H), 8.05 (d, *J* = 8.04 Hz, 1H), 9.31 (bs, 1H); ^13^C NMR (100 MHz, CDCl_3_) δ = 110.37, 114.02, 117.17, 123.14, 124.10, 129.43, 132.61, 135.23, 140.30, 148.92, 173.84

***N*-(4-Chlorophenyl)anthranilic acid**

Yield = 80 %

White powder

^1^H NMR (400 MHz, CDCl_3_) δ = 6.79 (t, *J* = 7.56 Hz, 1H), 7.16-7.21 (m, 3H), 7.32-7.39 (m, 3H), 8.05 (dd, *J* = 1.43 Hz and 1.42 Hz, 1H), 9.26 (bs, 1H); ^13^C NMR (100 MHz, CDCl_3_) δ = 110.66, 113.96, 117.65, 124.25, 129.03, 129.49, 132.67, 135.35, 138.95, 148.49, 173.62

***N*-(4-Methylphenyl)anthranilic acid**

Yield = 74 %

Pale brown crystal

^1^H NMR (400 MHz, CDCl_3_) δ = 2.35 (s, 3H), 6.70 (dd, *J* = 7.23 Hz and 7.23 Hz, 1H), 7.11-7.19 (m, 5H), 7.31 (quintet, 1H), 8.03 (dd, *J* = 1.01 Hz and 1.03 Hz, 1H), 9.22 (bs, 1H); ^13^C NMR (100 MHz, CDCl_3_) δ = 20.91, 109.89, 113.77, 116.68, 123.80, 130.00, 132.56, 134.06, 135.21, 137.51, 149.57, 174.01

***N*-(4-Isopropylphenyl)anthranilic acid**

Yield = 77 %

White powder

^1^H NMR (400 MHz, CDCl_3_) δ = 1.27 (d, *J* = 6.96 Hz, 6H), 2.92 (quintet, 1H), 6.72 (quintet, 1H), 7.16-7.33 (m, 6H), 8.03 (dd, *J* = 1.59 Hz and 1.58 Hz, 1H), 9.26 (bs, 1H); ^13^C NMR (100 MHz, CDCl_3_) δ = 24.08, 33.63, 109.89, 113.90, 116.71, 123.61, 127.34, 132.54, 135.16, 137.84, 145.07, 149.46, 173.44

***N*-(4-*tert*-Butylphenyl)anthranilic acid**

Yield = 87 %

White powder

^1^H NMR (400 MHz, CDCl_3_) δ = 1.34 (s, 9H), 6.72 (m, 1H), 7.18-7.40 (m, 6H), 8.02 (dd, *J* = 1.60 Hz and 1.64 Hz, 1H), 9.27 (bs, 1H); ^13^C NMR (100 MHz, CDCl_3_) δ = 31.44, 34.43, 110.00, 113.97, 116.76, 123.11, 126.28, 132.53, 135.14, 139.40, 147.40, 152.21, 170.15

***N*-(4-Decylphenyl)anthranilic acid**

Yield = 89 %

White powder

^1^H NMR (400 MHz, CDCl_3_) δ = 0.88 (t, *J* = 6.84 Hz, 3H), 1.27-1.32 (m, 16H), 2.60 (t, *J* = 7.73 Hz, 2H), 6.72 (quintet, 1H), 7.13-7.18 (m, 5H), 7.30-7.35 (m, 1H), 8.02 (dd, *J* = 1.48 Hz and 1.48 Hz, 1H), 9.25 (bs, 1H); ^13^C NMR (100 MHz, CDCl_3_) δ = 14.15, 22.71, 29.36, 29.55, 29.64, 31.60, 31.93, 35.45, 109.90, 113.87, 116.70, 123.65, 129.35, 132.56, 135.19, 139.20, 149.52, 173.60

***N*-(4-Methoxyphenyl)anthranilic acid**

Yield = 94 %

Yellow powder

^1^H NMR (400 MHz, CDCl_3_) δ = 3.83 (s, 3H), 6.69 (dd, *J* = 7.13 Hz and 7.07 Hz, 1H), 6.94 (t, *J* = 8.99 Hz, 3H), 7.19 (d, *J* = 8.84 Hz, 2H), 7.28-7.32 (m, 1H), 8.01 (dd, *J* = 1.33 Hz and 1.34 Hz, 1H), 9.14 (bs, 1H); ^13^C NMR (100 MHz, CDCl_3_) δ = 55.51, 109.32, 113.44, 114.70, 116.28, 126.40, 132.48, 132.94, 135.24, 150.46, 157.00, 173.40

***N*-(4-Hexyloxyphenyl)anthranilic acid**

Yield = 86 %

White powder

^1^H NMR (400 MHz, CDCl_3_) δ = 0.92 (t, *J* = 7.04 Hz, 3H), 1.33-1.39 (m, 4H), 1.44-1.50 (m, 2H), 1.76-1.83 (m, 2H), 3.97 (t, *J* = 6.58 Hz, 2H), 6.68-6.70 (m, 1H), 6.92 (dd, *J* = 2.16 Hz and 2.12 Hz, 2H), 6.94 (d, *J* = 8.70 Hz, 1H), 7.17 (dd, *J* = 2.18 Hz and 2.11 Hz, 2H), 7.28-7.32 (m, 1H), 8.01 (dd, *J* = 1.58 Hz and 1.58 Hz, 1H), 9.13 (bs, 1H); ^13^C NMR (100 MHz, CDCl_3_) δ = 14.05, 22.61, 25.74, 29.27, 31.60, 68.31, 109.26, 113.45, 115.29, 116.21, 126.39, 132.46, 132.70, 135.21, 150.52, 156.59, 173.38

***N*-(2-Trifluoromethylphenyl)anthranilic acid**

Yield = 74 %

White crystal

^1^H NMR (400 MHz, CDCl_3_) δ = 6.84 (quintet, 1H), 7.16-7.21 (m, 2H), 7.38 (quintet, 1H), 7.50 (t, *J* = 7.55 Hz, 1H), 7.58 (d, *J* = 8.11 Hz, 1H), 7.69 (d, *J* = 7.77 Hz, 1H), 8.09 (dd, *J* = 1.56 Hz and 1.58 Hz, 1H), 9.59 (bs, 1H); ^13^C NMR (100 MHz, CDCl_3_) δ = 111.87, 114.65, 118.36, 123.48, 124.04, 127.10, 127.15, 132.49, 132.69, 135.07, 139.10, 147.78, 173.13

***N*-(4-Trifluoromethylphenyl)anthranilic acid**

Yield = 79 %

White powder

^1^H NMR (400 MHz, CDCl_3_) δ = 6.87-6.91 (m, 1H), 7.33 (d, *J* = 8.40 Hz, 2H), 7.38 (dd, *J* = 0.92 Hz and 1.05 Hz, 1H), 7.42-7.46 (m, 1H), 7.59 (d, *J* = 8.49 Hz, 2H), 8.09 (dd, *J* = 1.52 Hz and 1.58 Hz, 1H), 9.45 (bs, 1H); ^13^C NMR (100 MHz, CDCl_3_) δ = 111.91, 114.94, 118.86, 120.70, 126.70, 132.77, 135.31, 143.93, 146.95, 172.90

***N*-(4-Nitrophenyl)anthranilic acid**

Yield = 67 %

Yellow powder

^1^H NMR (400 MHz, CDCl_3_) δ = 7.00-7.04 (m, 1H), 7.28-7.31 (m, 2H), 7.53-7.54 (m, 2H), 8.13 (d, *J* = 7.89 Hz, 1H), 8.19-8.23 (m, 2H), 9.66 (bs, 1H); ^13^C NMR (100 MHz, CDCl_3_) δ = 99.10, 113.61, 116.51, 118.17, 120.63, 125.81, 132.86, 135.28, 141.83, 144.97, 147.27, 170.5

***N*-(4-Fluoro-3-trifluoromethylphenyl)anthranilic acid**

Yield = 62 %

White powder

^1^H NMR (400 MHz, CDCl_3_) δ = 6.81-6.86 (m, 1H), 7.06-7.08 (m, 1H), 7.18-7.23 (m, 1H), 7.39-7.42 (m, 2H), 7.49 (dd, *J* = 2.68 Hz and 2.63 Hz), 8.07 (dd, *J* = 1.57 Hz and 1.57 Hz, 1H), 9.29 (bs, 1H); ^13^C NMR (100 MHz, CDCl_3_) δ = 110.86, 113.61, 117.89, 118.16, 121.96, 128.55, 128.63, 132.81, 135.61, 136.64, 148.40, 173.54
